# A parallel-group, multicenter randomized, double-blinded, placebo-controlled, phase 2/3, clinical trial to test the efficacy of pyridostigmine bromide at low doses to reduce mortality or invasive mechanical ventilation in adults with severe SARS-CoV-2 infection: the *P*yridostigmine *I*n *S*evere *CO*vid-19 (PISCO) trial protocol

**DOI:** 10.1186/s12879-020-05485-7

**Published:** 2020-10-16

**Authors:** Sergio Fragoso-Saavedra, David A. Iruegas-Nunez, Alejandro Quintero-Villegas, H. Benjamín García-González, Isaac Nuñez, Sergio L. Carbajal-Morelos, Belem M. Audelo-Cruz, Sarahi Arias-Martínez, Yanink Caro-Vega, Juan José Calva, Verónica Luqueño-Martínez, Alejandra González-Duarte, Brenda Crabtree-Ramírez, José C. Crispín, Juan Sierra-Madero, Pablo F. Belaunzarán-Zamudio, Sergio I. Valdés-Ferrer

**Affiliations:** 1 Programa de Estudios Combinados en Medicina (MD/PhD program), Universidad Nacional Autónoma de Mexico, Mexico City, Mexico; 2 Departamento de Neurología, Instituto Nacional de Ciencias Médicas y Nutrición Salvador Zubirán, Mexico City, Mexico; 3 Escuela Nacional de Medicina y Ciencias de la Salud, Instituto Tecnológico y de Destudios Superiores de Monterrey, Monterrey, Mexico; 4 Internal Medicine Residency Training Program, Instituto Nacional de Ciencias Médicas y Nutrición Salvador Zubirán, Mexico City, Mexico; 5 Departamento de Infectología, Instituto Nacional de Ciencias Médicas y Nutrición Salvador Zubirán, Mexico City, Mexico; 6 Departamento de e Inmunología y Reumatología, Instituto Nacional de Ciencias Médicas y Nutrición Salvador Zubirán, Mexico City, Mexico; 7 Division of AIDS, National Institute of Allergy and Infectious Diseases, National Institutes of Health, Rockville, MD USA; 8 Center for Biomedical Science, Feinstein Institute for Medical Research, Manhasset, NY USA

**Keywords:** COVID-19, SARS-Cov-2, Mortality, Invasive mechanical ventilation, Immunomodulation, Pyridostigmine, ACh, Inflammatory reflex, Placebo-controlled trial

## Abstract

**Background:**

Severe acute respiratory syndrome coronavirus 2 (SARS-CoV-2) infection, the causative agent of coronavirus disease 2019 (COVID-19), may lead to severe systemic inflammatory response, pulmonary damage, and even acute respiratory distress syndrome (ARDS). This in turn may result in respiratory failure and in death. Experimentally, acetylcholine (ACh) modulates the acute inflammatory response, a neuro-immune mechanism known as the *inflammatory reflex*. Recent clinical evidence suggest that electrical and chemical stimulation of the inflammatory reflex may reduce the burden of inflammation in chronic inflammatory diseases. Pyridostigmine (PDG), an ACh-esterase inhibitor (i-ACh-e), increases the half-life of endogenous ACh, therefore mimicking the inflammatory reflex. This clinical trial is aimed at evaluating if add-on of PDG leads to a decrease of invasive mechanical ventilation and death among patients with severe COVID-19.

**Methods:**

A parallel-group, multicenter, randomized, double-blinded, placebo-controlled, phase 2/3 clinical trial to test the efficacy of pyridostigmine bromide 60 mg/day P.O. to reduce the need for invasive mechanical ventilation and mortality in hospitalized patients with severe COVID-19.

**Discussion:**

This study will provide preliminary evidence of whether or not -by decreasing systemic inflammation- add-on PDG can improve clinical outcomes in patients with severe COVID-19.

**Trial registration:**

ClinicalTrials.gov NCT04343963 (registered on April 14, 2020).

## Background

Severe acute respiratory syndrome coronavirus 2 (SARS-CoV-2) infection, the causative agent COVID-19, may result in severe systemic inflammatory response. About one third of hospitalized patients with COVID-19 develop acute respiratory distress syndrome (ARDS) [1], while 17% require invasive mechanical ventilation associated to a high mortality rate [2]. The two main causes of death in patients with severe COVID-19 are respiratory and multiple-organ failure as a result of overwhelming inflammatory response [3, 4]. Therefore, patients with severe COVID-19 will theoretically benefit from therapeutic interventions that modulate the inflammatory response [5].

Pyridostigmine, an acetylcholinesterase inhibitor (i-ACh-e), increases acetylcholine (ACh) half-life by inhibiting its peripheral degradation. Pyridostigmine has been used for decades in the symptomatic treatment of myasthenia gravis [6] and as pre-exposure prophylaxis against nerve gas (chemical) warfare [7]. Pyridostigmine has well-characterized pharmacokinetic and safety profiles. Recently, pyridostigmine has been shown to reduce persistent inflammation in people living with HIV-1 infection [8–10]. The proposed anti-inflammatory effect occurs after the ACh binds to nicotinic receptors on the surface of immune cells and this interaction causes a decrease in the production of pro-inflammatory cytokines. This so-called inflammatory reflex, originally described in response to vagus nerve stimulation [11], leads to the release of ACh with a resulting reduction in acute [12] and chronic inflammation [13].

Our primary objective is to evaluate whether or not add-on pyridostigmine to best medical management of hospitalized COVID-19 patients will result in reduced need for invasive mechanical ventilation and death.

## Methods/design

### Aim, study design and settings

**Fig. 1:**
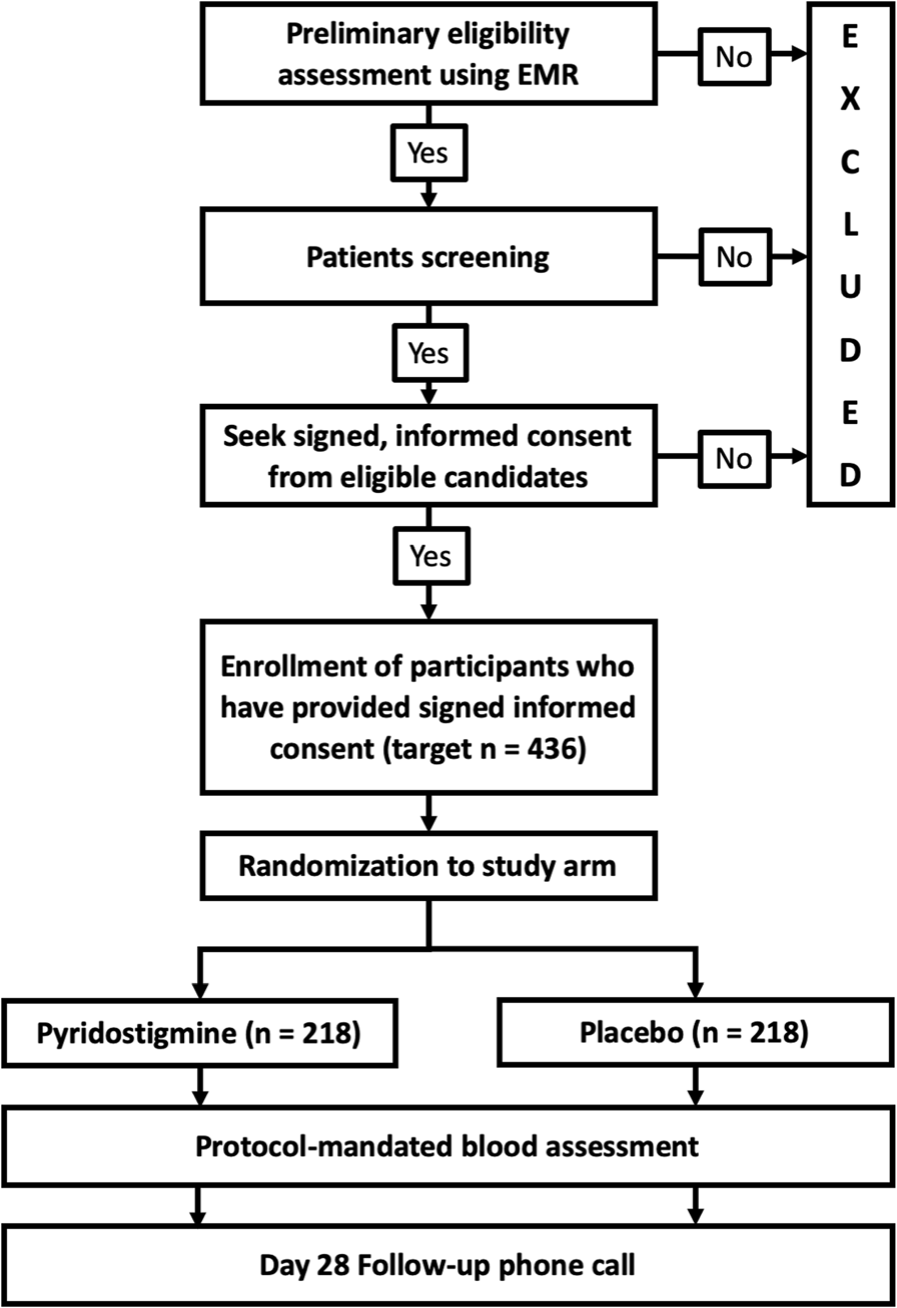
The PISCO trial (Pyridostigmine in Severe COVID-19) study design and schedule of follow-up visit

Our aim is to tests the efficacy of Pyridostigmine use as an immunomodulator to reduce the incidence of complications leading to critical illness or death in hospitalized adults with severe COVID-19. In order to test this, we propose a randomized, double-blinded, placebo-controlled trial. Participants will be randomly allocated in a 1:1 ratio to receive either oral pyridostigmine at a dose of 60 mg/day or a matching placebo for a maximum of 14 days in parallel groups. We will compare the need of invasive mechanical ventilation and fatality rates during the 28 days following randomization (Fig. 1). Unblinding will be permissible in case of severe adverse events at the request of the treating group of physicians, or at the request of the external Data and Safety Monitoring Board (DSMB).

The study is planned in two parts: a phase 2 aimed at determining security, followed by a phase 3 part aimed at evaluating the effect -or lack thereof- of pyridostigmine in patients with severe COVID-19. Phase 2 started recruiting on 5 May 2020. During the security (phase 2) part, we aimed at evaluating the safety and feasibility of the study and explore in a preliminary way the magnitude of the effect of the intervention. Safety was evaluated according to the frequency of outcomes as well as of reported adverse events. Enrollment for the security phase was limited to patients hospitalized at Instituto Nacional de Ciencias Médicas y Nutrición Salvador Zubirán (INCMNSZ) in Mexico City. On 4 July 2020, a pre-appointed DSMB performed an *ad interim* analysis after the first 44 participants (10% of the calculated sample) had been recruited and, as results derived from this security part indicated that pyridostigmine was not associated with an increased frequency of outcomes or adverse events (safety outcome), the DSMB recommended to proceed to a multi-center, phase 3 trial. During this ongoing phase 3 component of the RCT, the primary outcome to be evaluated will be a composite outcome including 1) the requirement of invasive mechanical ventilation, 2) an increase in the SOFA scale ≥2 points, or 3) death.

The DSMB also suggested to repeat an *ad interim* analysis every time a 10%-recruiting milestone is reached.

### Study population

We are including adult (≥18-year-old), hospitalized patients with confirmed SARS-CoV-2 infection based on a positive RT-PCR test for SARS-CoV-2 RNA in a respiratory specimen (nasopharyngeal or nasal swab) and an imaging study compatible with pneumonia, and at least one high-risk criteria of death (see Table 1).

**Table 1:** Inclusion criteria

**1)** Adults (≥18 years old),	
**2)** Confirmed SARS-CoV-2 infection based on a positive RT-PCR test	
**3)** Requiring in-hospital care	
**4)** Imaging study compatible with pneumonia,	
**5)** At least one of the following criteria	
**a).** Dyspnea	
b). Lung infiltrates occupying > 50% of lung fields by CT scan	
c) PaO2/FiO2 ratio < 300 mmHg	
d). Peripheral oxygen saturation (SpO2) < 90% while breathing room air, a ≥ 3% drop in baseline SpO2, or the need of increased flow rates of supplemental oxygen in the case of chronic hypoxia; and the need for supplemental oxygen therapy according to the treating medical team’s judgment.	
e). Alteration of one or more of the following laboratory parameters	
**•** D-dimer > 1 μg/mL	
**•** Ferritin level > 300 ng/mL	
**•** C-reactive protein (CRP) > 3 mg/L	
**•** Lactate dehydrogenase (LDH) > 245 U/L	
**•** Lymphopenia, defined as < 800 lymphocytes/uL	
**•** Creatine kinase (CK) level > 800 IU/L	

Exclusion criteria include one or more of the following: allergy to pyridostigmine; pregnancy or breastfeeding status; concomitant autoimmune disease; diagnosed immunodeficiencies (including HIV infection); need for mechanical ventilation, admission to the ICU, or meeting criteria for septic shock before providing signed, informed consent; inability to receive orally or enterally administered drugs; use of immunosuppressants or immune-modulators (including chemotherapy and corticosteroids) in the preceding 28-day period unless recommended by the treatment medical team as part of the therapeutic approach for SARS-CoV-2 infection; and participation in clinical trials of any kind in the previous 28 days.

### Procedures

#### Randomization

Participants will be randomized in a 1:1 ratio, with parallel assignment to receive either placebo or pyridostigmine as an add-on medical treatment to the best medical care available for severe COVID-19 in participating centers. The block-randomization process will be performed using the publicly available online resource (www.randomizer.org).

#### Intervention

**Fig. 2:**
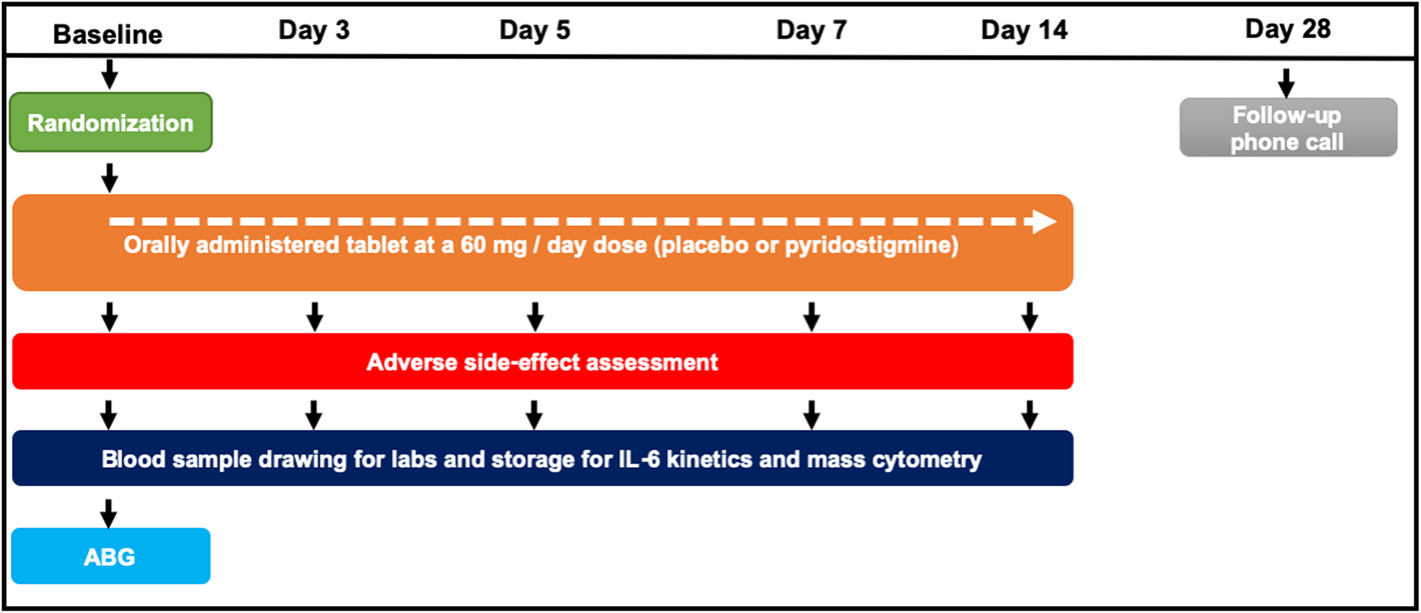
Protocol Activities. All timepoints are counted from enrollment (Baseline). Arrows indicate specific actions to be performed at each predefined timepoint. Patients who are discharged from hospital before day 14 are not required to return for blood sampling. Abbreviations: ABG: arterial blood gases; IL-6: interleukin 6

Patients in the treatment group will receive Pyridostigmine Bromide, 60 mg/day per os. Participants randomized to the control group will receive matching placebo (identical in appearance) made of pharmaceutical grade starch. Participants will be receiving the assigned intervention until the occurrence of either 1) any of the pre-specified outcomes; 2) hospital discharge; or 3) a maximum period of 14 in-hospital days (Fig. 2).

### Outcomes and definition of variables

The primary outcomes are a composite of requirement of invasive mechanical ventilation, an increase of ≥2 points in the SOFA scale, or all-cause mortality, during the 28-day period following enrollment; and, 2) safety of the study drug. The secondary outcome is the change in interleukin (IL)-6 levels (Δ IL-6) between baseline samples and those taken on days 3, 5, 7, and 14 (for an outline of the protocol, please refer to Fig. 2; for the timeline of interventions and measurements, please refer to Table 2).

**Table 2:** Scheduled protocol activities

	Baseline	Day 3	Day 5	Day 7	Day 14	Day 28
**Eligibility preliminary assessment**	X					
**Eligibility confirmation**	X					
**Informed consent & enrollment**	X					
**Study medication supply**	X					
**Adverse-effect assessment**^**a**^	X	X	X	X	X	
**Laboratory evaluations**^**a**^						
**• D-dimer**	X	X	X	X	X	
**• Ferritin**	X	X	X	X	X	
**• CPK**	X	X	X	X	X	
**• Fibrinogen**	X	X	X	X	X	
**• Creatinine**	X	X	X	X	X	
**• Bilirubin**	X	X	X	X	X	
**• CBC**	X	X	X	X	X	
**• ABG**	X					
**Storage of samples for posterior pooled analysis**^**a**^						
**• IL-6 measurement**	X	X	X	X	X	
**• Mass cytometry**	X	X	X	X	X	
**Follow-up phone call**						X

^a^Blood sampling will be performed only while participants are hospitalized. Protocol does not require participants to return for further blood sampling after hospital discharge. Abbreviations: *CBC* Complete blood count, *ABG* Arterial blood gases, *IL-6* Interleukin 6

We will collect demographic information from participants at baseline, including age, sex assigned at birth, presence of comorbidities which will include diabetes mellitus, systemic arterial hypertension, obesity, cardiovascular disease, and lung disease, and other chronic medical conditions from the clinical charts. Safety of the intervention will be actively evaluated by daily interrogation of the following common adverse effects of pyridostigmine [14]: abdominal pain or cramps; diarrhea; nausea, vomiting, or both; hypersalivation/drooling; urinary incontinence; muscle weakness or fasciculations; and, blurred vision. On day 28, patients will be contacted by telephone to assess their vital and functional status (Fig. 2; Table 2). All collected data will be safeguarded on a coded database with access limited to project investigators. Only the principal investigators and the DSMB will have access to the final trial dataset. The final results will be published for generalized access, regardless of the outcome.

### Study centers

Currently, recruiting for this study is undergoing at, *Instituto Nacional de Ciencias Médicas y Nutrición Salvador Zubirán* (INCMNSZ)*,* and *Instituto Nacional de Cardiología Ignacio Chávez*, two COVID-19-designated Hospitals in Mexico City, Mexico.

### Sample size

#### First (security) phase

We estimate that a sample size of 40 participants (20 in each group) would produce a one-sided 80% confidence limit that would exclude us finding a 10%-point difference that would be statistically significant in the complete trial [15]. However, calculating a 10% loss, we will recruit 44 participants for this part of the study.

#### Second phase

We estimate that a sample size of 436 participants (218 per group) estimating a 10% reduction in the occurrence of the primary outcome in the intervention group to be clinically significant. Based on recent evidence from China, we estimate that 25% of patients hospitalized with severe SARS-CoV-2 infection will develop complications leading to the need of invasive mechanical ventilation or death [16] Accordingly, we estimate that this sample size will allow us to identify with an 80% power a reduction in the need of invasive mechanical ventilation or death of 10% in the group receiving pyridostigmine in comparison with the group on placebo, using a two-sided *t*-test at the 0.05 significance level.

### Statistical considerations

Primary analysis will be performed by intention-to-treat analysis comparing the proportion of outcome events between groups using *X*^*2*^ test. We will also compare point estimates and its corresponding confidence intervals between groups. In a secondary analysis, we will use multivariate logistic regression models to explore variables associated with the primary outcomes.

### Study status

This is an ongoing study. Recruiting started on 5 May 2020; at submission, we have recruited 86 participants. No results have been made available, and the therapeutic arms remain double blinded. Therefore, no results have been submitted for publication or published.

## Discussion

Here, we propose to evaluate the potential usefulness of pyridostigmine as add-on therapy to best medical care of patients admitted to a hospital due to severe COVID-19. Recent evidence indicates that between 25 and 33% of patients hospitalized for COVID-19 required care in intensive care units (ICU) for severe hypoxemia. The reported mortality in those first cases that required hospital management is 15%, but in those with severe disease, the reported mortality is between 38 and 49% [2, 16], and we assume that it will be similar elsewhere. Severity and mortality of COVID-19 appear to be mediated not by infection, but by the disproportionate inflammatory response of the host. Hence, finding novel immunomodulatory strategies is a promising strategy to reduce severity and mortality of COVID-19. Furthermore, the repurposing of drugs with well characterized safety profiles and readily available production lines, might lead to faster development of anti-COVID-19 therapies if proven efficacious in well-designed, randomized clinical trials.

In mammals, the central nervous system has mechanisms to control the inflammatory response. During inflammatory states, the vagus nerve can inhibit the synthesis and release of inflammatory cytokines [17], thereby reducing both local damage and mortality secondary to severe systemic inflammation in murine models as diverse as sepsis, ischemia and re-perfusion damage, or obesity [18–21]. The vagus nerve can be stimulated electrically and chemically. Chemical stimulation using cholinergic agonists has shown promising effects in murine and cellular models of inflammation [12, 21].

Acetylcholine esterase inhibitors (i-ACh-e) are a family of drugs used regularly by millions of patients, including older adults with Alzheimer disease and other dementias, as well as in patients with myasthenia gravis and dysautonomia [6, 22–25]. These drugs inhibit the enzymatic degradation of endogenous ACh, resulting in greater bioavailability and, therefore, an increase in the possibility of binding to both nicotinic and muscarinic receptors. In addition to the approved uses of i-ACh-e in human pathology, there is evidence in various murine models of their efficacy in experimental sepsis and severe inflammatory response [12, 19, 21], suggesting that i-ACh-e drugs have a potential immunomodulatory effect in patients with severe systemic inflammatory response syndrome. Pyridostigmine, an acetylcholinesterase inhibitor, has been previously shown to decrease inflammation in people living with human immunodeficiency virus (HIV) infection [8–10]; therefore, it is possible that Pyridostigmine may lead to a decrease in the production of pro-inflammatory cytokines in patients with COVID-19 at high risk of severe disease.

Regarding safety concerns, at the proposed dose of Pyridostigmine, the rate of adverse events is less than 5–6% with no reported serious adverse effects [14]. From this perspective, we consider that pyridostigmine can function as an immunomodulator and reduce morbidity and mortality in these patients. The reduction in the frequency of the need for mechanical ventilation would contribute to reducing mortality and the demand for these services.

## Data Availability

The final datasets will be made publicly available in the final manuscripts, supplemental materials, or public repositories. All protocol documents are available in Spanish upon reasonable requests.

## Abbreviations

SARS-CoV-2: Severe acute respiratory syndrome coronavirus 2
COVID-19: Coronavirus Associated Disease 2019
ARDS: Acute Respiratory Distress Syndrome
MV: Invasive Mechanical Ventilation
ACh: Acetylcholinesterase Inhibitor
HIV-1: Human Immunodeficiency Virus Type 1
PISCO trial: Pyridostigmine in Severe COVID-19
INCMNSZ: Instituto Nacional de Ciencias Médicas y Nutrición Salvador Zubirán
SOFA: Sequential Organ Failure Assessment score
ICU: Intensive Care Unit
IL-6: Interleukin-6
i-ACh-e: Acetylcholine esterase inhibitors
